# Incidence de l'epilepsie après un accident vasculaire cerebral à Parakou en 2014

**DOI:** 10.11604/pamj.2019.32.69.16897

**Published:** 2019-02-07

**Authors:** Adoukonou Thierry, Accrombessi Donald, Agbétou Mendinatou, Houinato Dismand

**Affiliations:** 1 Unité d'Enseignement et de Recherche de Neurologie, Faculté de Médecine, Université de Parakou, Benin; 2 Service de Neurologie, CHU Parakou, Benin; 3 Unité d'Enseignement et de Recherche de Neurologie, FSS Université d'Abomey-Calavi, Benin

**Keywords:** Incidence, épilepsie, accident vasculaire cérébrale, incidence, epilepsy, stroke

## Abstract

**Introduction:**

Les accidents vasculaires cérébraux (AVC) constituent une des principales causes acquises de l'épilepsie de l'adulte mais peu de données sont disponibles sur l'incidence de l'épilepsie après un AVC en Afrique. L'objectif de cette étude était d'estimer l'incidence de l'épilepsie chez les patients victimes d'AVC à Parakou.

**Méthodes:**

Il s'est agi d'une étude de type cohorte dynamique ayant inclus 203 patients victimes d'AVC et hospitalisés dans le service de neurologie du CHU de Parakou. Les patients aux antécédents d'épilepsie étaient exclus du suivi. Les patients étaient suivis sur une période de 21mois allant du 1^er^ Janvier 2013 au 30 Septembre 2014. L'épilepsie était définie selon les critères de la Ligue Internationale contre l'Epilepsie.

**Résultats:**

Ils étaient âgés de 18 à 99 ans avec une moyenne de 58,4 ans ± 14,2 ans. Le délai moyen de consultation après l'installation des symptômes était de 54,3h (+/-112,9h). Les AVC ischémiques représentaient 45,8%, les hémorragiques 31% et 23,2% d'indéterminés. L'incidence cumulée de l'épilepsie était de 17. La densité d'incidence de l'épilepsie était de 9,8 pour 100 personnes-années. Les facteurs associés à la survenue de l'épilepsie étaient le niveau d'instruction, la durée d'hospitalisation et le score de BARTHEL.

**Conclusion:**

L'incidence de l'épilepsie reste très élevée et la prise en compte des facteurs associés dans les stratégies de prise en charge pourrait permettre de réduire sa charge globale.

## Abstract (en)

**Introduction:**

Cerebrovascular accidents (strokes) are one of the main acquired causes of epilepsy in adults, but little data are available on the incidence of epilepsy after a stroke in Africa. This study aims to estimate the impact of epilepsy in stroke patients in Parakou.

**Methods:**

We conducted a dynamic cohort study including 203 stroke patients hospitalized in the Department of Neurology at the University Hospital of Parakou. Patients with a history of epilepsy were excluded from the follow-up. Patients were monitored over a 21-month period from 1 January 2013 to 30 September 2014. Epilepsy was defined according to the International League Against Epilepsy criteria.

**Results:**

Patients were aged 18-99 years, with an average age of 58.4 ± 14.2 years. The median of consultation time after symptoms was 54,3 h (+/-112,9h). Ischemic strokes accounted for 45.8%, haemorrhagic strokes 31% and indeterminate strokes 23.2%. The cumulative impact of epilepsy was 17. The incidence density rate of epilepsy was 9.8 per 100 person-years. Factors associated with the occurrence of epilepsy were the educational level, the duration of hospitalization and Barthel score.

**Conclusion:**

The incidence of epilepsy is very high; hence, taking into consideration the factors associated with it during the implementation of a treatment strategy may reduce its overall load.

## Introduction

Les accidents vasculaires cérébraux constituent un véritable problème de santé publique en Afrique [1]. Sa charge est importante à travers la mortalité et son coût socio-économique assez élevés [1]. Les comorbidités viennent alourdir cette charge. Parmi celles-ci l'épilepsie est l'une des plus fréquentes et les plus handicapantes. L'épilepsie est l'une des maladies neurologiques les plus fréquentes. Sa prévalence en Afrique est estimée entre 1 et 3% [2]. A Parakou une étude réalisée en 2011 a retrouvé une prévalence de 0,7% chez les adultes de plus de 15ans [3]. Les AVC constituent l'une des causes les plus fréquentes de l'épilepsie symptomatique de l'adulte [4]. L'incidence de crise épileptique après AVC est estimée dans une méta-analyse à 7% et celle de l'épilepsie de 5% [5]. Dans cette méta-analyse l'incidence est plus élevée en cas d'AVC hémorragique et en cas de localisation corticale [5]. L'épilepsie vasculaire est définie par la récurrence de crise épileptique après un AVC [6]. Une crise unique après un AVC n'est pas considérée comme une épilepsie. La fréquence de l'épilepsie après un AVC est de 2% au Bénin [7] et 1,98% au Burkina [8]. Toutefois des études d'incidence de l'épilepsie après un AVC en Afrique subsaharienne sont rares étant donnée les difficultés de suivi à long terme et de réalisation d'études de cohortes dans cette région. L'objectif de cette étude était d'étudier l'incidence des crises épileptiques et de l'épilepsie chez les patients victimes d'accident vasculaire cérébral à Parakou.

## Méthodes

**Cadre d'étude:** L'étude s'est déroulée à Parakou à 425 Km au Nord-Est de Cotonou au Bénin plus précisément dans le Centre Hospitalier Universitaire de Parakou. Cet hôpital dispose d'un service de neurologie disposant de deux neurologues dont un neurologue vasculaire, d'un service de réanimation avec 3 réanimateurs et y travaillent également deux neurochirurgiens, un cardiologue. Il dispose aussi d'un service de kinésithérapie. Parakou dispose de deux unités de scanner et de centre d'explorations cardiovasculaires (ECG, échographie cardiaque, doppler des vaisseaux du cou). Le service de neurologie et celui de la réanimation reçoivent les sujets ayant un accident vasculaire cérébral. Le suivi après AVC est effectué par les neurologues.

**Type d'étude:** Nous avons réalisé une étude observationnelle de type cohorte qui s'est déroulée du 1^er^ Janvier 2013 au 30 Septembre 2014. La cohorte était dynamique. L'étude comportait deux phases: 1^ère^ phase du 1^er^Janvier au 31 Décembre 2013 et 2^ème^ phase suivi des survivants jusqu'au 30 Septembre 2014, tous les survivants d'accident vasculaire cérébral ont été inclus.

**Critères d'inclusion:** Etre hospitalisé dans l'une des structures ci-dessus énumérées pour un AVC dans la période d'étude et avoir donné son consentement éclairé pour participer à l'étude.

**Critère d'exclusion:** Tout patient connu épileptique ou ayant fait au moins une crise épileptique avant l'AVC.

**Critère de jugement:** Etait considérée comme crises épileptique toute manifestation paroxystique survenue après l'AVC décrite comme telle rapportée et confirmée par le neurologue, le questionnaire d'investigation de l'épilepsie en milieu tropical de l'Institut d'Epidémiologie et de Neurologie Tropicale [9] a été utilisé et une réponse “oui” à une des cinq questions de dépistage avec confirmation du diagnostic par un neurologue était considérée comme une crise épileptique. Les sujets ayant fait deux crises épileptiques confirmées sont considérés comme épileptiques.

**Echantillonnage:** Nous avons procédé à un recrutement exhaustif de tous les sujets remplissant les critères d'inclusion. La cohorte étant dynamique; en début de suivi 85 sujets étaient inclus (P0) et à la fin de l'étude on notait 131 survivants (P1). Entre le début de l'étude et la fin du suivi soit 21 mois, au total 203 sujets sont suivis.

**Collecte des données:** Après inclusion de chaque sujet un suivi périodique téléphonique était fait par une infirmière chaque mois pour renseigner le statut (vivant ou décédé de chaque sujet). Les sujets décédés étant exclus du suivi. Les patients, après leur sortie de l'hôpital, étaient systématiquement reçus en consultation de suivi post-AVC par le neurologue à 2 semaines, 1 mois, 3 mois, 6 mois, 1 an et tous les 6 mois. Lors de cet entretien (par l'infirmière) ou au cours des consultations de suivi le questionnaire de dépistage de l'épilepsie était utilisé et la notion de crise épileptique était renseignée. Pour les patients aphasiques ou incapables de répondre aux questions un proche était questionné. Les données sur l'AVC pendant l'hospitalisation et le devenir étaient collectées. Il s'agit des informations sociodémographiques (âge, sexe, niveau d'instruction, ethnie), les facteurs de risque vasculaire (hypertension artérielle, diabète, tabagisme, obésité) l'état clinique à l'admission (NIHSS, score de Glasgow, poids, taille, Température, pression artérielle) données paracliniques (type d'AVC, territoire concerné), les complications relatives à l'AVC (pneumopathie, infection urinaire, phlébite, embolie pulmonaire), et les facteurs évolutifs (score de RANKIN, Score de BARTHEL).

**Traitement et analyse des données:** Toutes les données collectées ont été traitées, saisies et analysées avec les logiciels SPSS version 17.0 et Excel 2013 (windows 2007). Les variables quantitatives ont été exprimées en moyenne avec un écart-type, les données qualitatives en pourcentage. Pour déterminer la densité d'incidence de l'épilepsie, le nombre d'événements observés a été divisé par le nombre de personnes-temps à risque. Ce nombre est estimé comme dans le cas d'une cohorte dynamique et est égal à (P0 +P1-m)/2 où Po est l'effectif de la cohorte au départ et P1 l'effectif à la fin et m le nombre de décès et est exprimée en personnes-année. Les sujets décédés étant considérés comme n'étant plus à risque. La cohorte étant considérée comme dynamique. Ensuite, nous avons procédé à des analyses bivariées entre la variable dépendante et les variables indépendantes. Pour ces analyses seules les incidences cumulées étaient considérées. Les différentes fréquences ont été comparées à l'aide du test de chi² de Pearson ou du test exact de Fisher selon le cas. La comparaison de moyenne était effectuée avec le test t de *Student.* La différence était considérée comme statistiquement significative lorsque p<0,05.

## Résultats

Au total 203 sujets étaient inclus dans notre cohorte dont 113 hommes (55,7%). Ils étaient âgés de 18 ans à 99 ans avec une moyenne de 58,4+/-14,2ans. Les caractéristiques de ces sujets sont résumées dans le Tableau 1. Sur 203 sujets, 152 (74,9%) n'avaient aucune couverture médicale (assurance). Le délai moyen d'admission après le déficit était de 54,3 heures (+/-112,9) avec des extrêmes allant de 20 minutes à 720 heures. 52 patients (25,6%) étaient admis dans un délai de 3h après l'installation des symptômes; 156 (76,8%) ont pu réaliser le scanner cérébral. L'hypertension artérielle (68,5%), l'alcoolisme (21,7%) et le diabète (16,7%) étaient les facteurs de risque les plus retrouvés. Le score NIHSS moyen à l'admission était de 12,1 ± 5,8 avec des extrêmes de 1 et 32. Les AVC ischémiques représentaient 45,8%, les hémorragiques 31,0% et les indéterminés (ceux n'ayant pas réalisé le scanner cérébral) 23,2%. Parmi les victimes d'AVC hémorragique prédominait l'hématome profond (81%); les localisations lobaires ne représentaient que 19% tandis que chez les victimes d'AVC ischémique le territoire le plus touché était celui de l'artère sylvienne (73,1%) et 10,8% dans le territoire du cérébral antérieur. Aussi sur les 156 sujets ayant un scanner cérébral 73 (46,8%) ont une localisation corticale ou superficielle de leur AVC. Sur le plan évolutif 72 patients étaient décédés soit un taux global de mortalité de 35,5%. Une bonne observance thérapeutique était observée chez 111 (54,7%). Pendant la phase aiguë six patients avaient présenté une crise d'épilepsie soit une fréquence de 3,0%. Mais durant toute la période de suivi de la cohorte 22 personnes (10,8%) avaient présenté au moins une crise épileptique et 17 avaient une épilepsie confirmée (par le neurologue). Le nombre de personnes à risque N est déterminée par la formule (cohorte dynamique)

**Tableau 1:** Caractéristiques des sujets dans l’enquête épilepsie post-AVC, Parakou 2014

	Population N (%)	Epilepsie N (%)	P value
**Age (ans)**			0,1
15 – 29	3 (1,5)	0 (0,0)	
30 – 44	20 (9,8)	3 (15,0)	
45 – 59	84 (41,4)	5 (5,9)	
60 – 74	67 (33,0)	6 (9,0)	
75 – 89	26 (12,8)	3 (11,5)	
90 et plus	3 (1,5)	0 (0,0)	
**Sexe**			0,1
Hommes	113 (55,7)	12 (10,6)	
Femmes	90 (44,3)	5 (5,6)	
**Niveau d’instruction**			0,03
Non instruit	124 (61,1)	8 (6,5)	
Primaire	22 (10,8)	0 (0,0)	
Secondaire	42 (20,7)	8 (19,0)	
Supérieur	15 (7,4)	1 (6,7)	
**Profession**			0,7
Cadres et assimilés	27 (13,3)	4 (14,8)	
Artisans/Commerçants	25 (12,3)	3 (12,0)	
Femmes au foyer	61 (30,0)	4 (6,6)	
Ouvriers/cultivateurs	39 (19,2)	3 (7,7)	
Retraités	25 (12,3)	2 (8,0)	
Autres	16 (7,9)	1 (6,2)	
**Statut matrimonial**			0,4
Vit en couple	162 (79,8)	13 (8,0)	
Vit seul	41 (20,2)	4 (9,7)	
**Type d’AVC**			
Ischémique			
93 (45,8)			
12 (12,9)	0,07		
Hémorragique	63 (31,0)	4 (6,2)	
Indéterminé	47 (23,2)	1 (2,1)	

AVC= Accident vasculaire cérébral

N=(P0+P1-M)2

« Po » le nombre de personnes au début de la cohorte (Po= 85) , « P1 » le nombre de personne à la fin du suivi (P1= 131), « M » le nombre de personne ayant présenté une épilepsie (M=17), N= (131+85-17)/2. Le nombre de personnes temps à risque (N'), N'= (N× le nombre de mois) ÷ 12 N'= (99,5× 21) ÷ 12= 174,1 personnes-année à risque. L'incidence de l'épilepsie est donc de 9,8 pour 100 personnes-années (M/N'). Les facteurs associés à la survenue d'épilepsie sont résumés dans les Tableau 1 et Tableau 2.

**Tableau 2:** Fréquence de l’épilepsie suivant certaines caractéristiques quantitatives des sujets ayant un AVC, Parakou 2014

Variables	Epilepsie présente	Epilepsie absente	P value
**Age (années)**			0,8
Moyenne (±ET)	58,2 (±15,5)	60 (±12,2)	
[Min-Max]	[30-89]	[40-85]	
**NIHSS Initial** Moyenne	12,6 (± 5,9)	12,6 (± 5,9)	0,7
(±ET)	[3-21]	[2-23]	
[Min-Max]			
**Barthel** Moyenne (±ET)	45,1 (± 18)	63,9 (±21,1)	0,00
[Min-Max]	[10-80]	[15-100]	
**Glasgow** Moyenne (±ET)	13,3 (± 3,4)	12,9 (±3,1)	0,7
[Min-Max]	[7-15]	[3-15]	
**IMC**			0,4
Moyenne (±ET)	24,48 (±4,8)	24,6	
[Min-Max]	[19,8-34,1]	[14,7- 33,7]	
**Durée d’hospitalisation**			0,03
Moyenne (±ET)	9,6 (± 5,8)	12,6 (±11,9)	
[Min-Max]	[2-24]	[2-43]	
**PAS**			0,8
Moyenne (±ET)	165,8±31,4	170,7 (±39,7)	
[Min-Max]	[110-240]	[100-256]	
**PAD**			0,2
Moyenne (±ET)	99±19,7	112,9 (±26,3)	
[Min-Max]	[60-145]	[70-160]	

IMC: Indice de Mace Corporel, PAS : pression artérielle systolique, PAD : pression artérielle diastolique ; ET : écart-type ; Min : minimum ; Max : maximum

## Discussion

Le principal objectif de cette étude était d'étudier l'incidence des crises épileptiques et de l'épilepsie chez les patients victimes d'accident vasculaire cérébral à Parakou. A notre connaissance, il s'agit de la première étude dans le septentrion du Bénin sur le sujet. Pour atteindre cet objectif, nous avions réalisé une étude de type cohorte dynamique en deux phase: une première phase d'inclusion et une deuxième de suivi des survivants. Les données ont été collectées à l'aide d'une fiche d'enquête préétablie à partie de la revue de littérature. Le questionnaire était expliqué au patient ou à l'entourage et leur consentement était obtenu. Nous avions donc utilisé une méthodologie rigoureuse et estimons que cette étude a une bonne validité. Dans notre étude, nous avions constaté que le taux de mortalité après un AVC était de 35,5%. Ce résultat est similaire à la mortalité après AVC dans le monde 35,6% [10]. Ceci est sans doute dû au fait que les AVC constituent une pathologie grave avec une charge importante. Elle pourrait également s'expliquer par le fait que dans notre étude, 74,9% des patients n'ont pas une couverture sanitaire donc ne peuvent s'offrir des soins spécifiques. Des taux élevés ont été rapporté dans la sous-région notamment au Nigéria 50% par Danesi *et al.* et 45% par Farner *et al.* [11, 12]. Cela pourrait s'expliquer par le fait que malgré les ressources dont dispose le Nigéria, le système sanitaire n'est pas encore bien régulé.

Des taux de mortalité plus faible ont été rapportés dans les pays du nord notamment en France où elle était de 13,1% à un an en 2013 [13]. Cela s'expliquerait par le fait que la France de ressources illimitées pour la prise en charge des patients victimes d'AVC. Il ressort de cette étude que l'incidence de l'épilepsie après un AVC est de 9,8 pour 100 personnes-années. Des incidences inférieures au notre ont été rapportées dans la littérature. C'est le cas de la France où Rossi *et al.* [14] rapportait en 2013 une incidence de 4 pour 100 personne-années, et de la Taïwan où Chen *et al.* [15] avait trouvé en 2013 une incidence de 2,6%. Ces taux inférieurs pourraient s'expliquer par le fait que ces pays disposent de moyen de prise en charge notamment la thrombolyse dans le cadre de l'accident vasculaire cérébrale ischémique pour assurer la revascularisation cérébrale limitant ainsi les complications et même la mortalité péri accident vasculaire cérébral. Dans cette étude, le type d'AVC n'était pas associé à la survenue d'une épilepsie mais nous avions constaté que dans la population des épileptiques, les patients ayant fait un AVC ischémique étaient majoritaires. Ce constat n'est pas le même que dans la littérature puisque les différents auteurs [16-18] rapportaient que dans la survenue d'épilepsie après un AVC c'est l'hématome intra parenchymateux et de siège corticale qui non seulement était associé mais était prédominant. Notons que dans notre étude les patients qui avaient un infarctus cérébral étaient majoritaires 45,9% avec une prédominance de l'artère sylvienne superficielle (73,1%) tandis que les patients victimes d'AVC hémorragique avaient pour la plupart un hématome profond (81%) donc moins à risque de faire une crise convulsive que les patients ayant fait un AVC ischémiques qui étaient en majorité corticale.

Nous avions constaté au terme de ce travail que le niveau d'instruction était significativement associé à la survenue d'une épilepsie après un AVC. Il n'existe pas de substratum anatomo-physiologique expliquant cet état de chose mais nous pensons que cela pourraient être en rapport avec l'ignorance de la population des facteurs de risques vasculaires, des signes d'un AVC et de l'urgence que cela représente. Ces derniers après avoir fait un AVC, font d'abord une auto prise en charge à domicile avant de transiter par des cabinets non spécialisés pour une orientation vers le spécialiste justifiant ainsi les longs délais d'admission (54,3 heures +/- 112,9) dans notre contexte. La durée d'hospitalisation était associée de manière significative à l'AVC dans cette étude. Les patients épileptiques avaient une durée moyenne d'hospitalisation inférieure à ceux n'ayant pas faire d'épilepsie (9,6 (± 5,8) contre 12,6 (±11,9)). Deux raisons pourraient expliquer ce constat: le mode de sortie des patients qui rentre pour la plupart contre avis médical (dans notre étude 19,8%) et les patients AVC hémorragique qui restaient deux semaines au moins en hospitalisation période ou la récidive est fréquente.

## Conclusion

The incidence of epilepsy remains very high and taking into account associated factors in management strategies could reduce its overall burden. Keywords: incidence, seizure, epilepsy, stroke

### Etat des connaissances actuelles sur le sujet

- Les accidents vasculaires cérébraux constituent une des principales causes de l'épilepsie acquise de l'adulte;
- la localisation de l'AVC ainsi que la survenue de crises précoces en constituent les meilleurs prédicteurs.

### Contribution de notre étude à la connaissance

- De rare études d'incidence sur l'épilepsie post-AVC en Afrique ont été menées avec un suivi à plus de deux crises et dégageant des facteurs tels le niveau d'instruction, la durée d'hospitalisation et le BARTHEL;
- L'incidence étant un meilleur indicateur épidémiologique de la fréquence d'un phénomène de santé, les données de notre étude pourraient refléter la réelle fréquence de l'épilepsie après un AVC en Afrique subsaharienne.

## Conflits d'intérêts

Les auteurs ne déclarent aucun conflit d'intérêts.
